# Oral glutathione supplementation drastically reduces *Helicobacter*-induced gastric pathologies

**DOI:** 10.1038/srep20169

**Published:** 2016-02-02

**Authors:** Ellen De Bruyne, Richard Ducatelle, Dennis Foss, Margaret Sanchez, Myrthe Joosten, Guangzhi Zhang, Annemieke Smet, Frank Pasmans, Freddy Haesebrouck, Bram Flahou

**Affiliations:** 1 Department of Pathology, Bacteriology and Avian Diseases, Faculty of Veterinary Medicine, Ghent University, Salisburylaan 133, 9820 Merelbeke, Belgium; 2 Zoetis, Kalamazoo, MI 49007 USA

## Abstract

*Helicobacter* (*H.*) *suis* causes gastric pathologies in both pigs and humans. Very little is known on the metabolism of this bacterium and its impact on the host. In this study, we have revealed the importance of the glutamate-generating metabolism, as shown by a complete depletion of glutamine (Gln) in the medium during *H. suis* culture. Besides Gln, *H. suis* can also convert glutathione (GSH) to glutamate, and both reactions are catalyzed by the *H. suis* γ-glutamyltranspeptidase (GGT). Both for *H. pylori* and *H. suis*, it has been hypothesized that the degradation of Gln and GSH may lead to a deficiency for the host, possibly initiating or promoting several pathologies. Therefore the *in vivo* effect of oral supplementation with Gln and GSH was assessed. Oral supplementation with Gln was shown to temper *H. suis* induced gastritis and epithelial (hyper)proliferation in Mongolian gerbils. Astonishingly, supplementation of the feed with GSH, another GGT substrate, resulted in inflammation and epithelial proliferation levels returning to baseline levels of uninfected controls. This indicates that Gln and GSH supplementation may help reducing tissue damage caused by *Helicobacter* infection in both humans and pigs, highlighting their potential as a supportive therapy during and after *Helicobacter* eradication therapy.

Non-*Helicobacter* (*H.*) *pylori Helicobacter* (NHPH) species have been found colonizing the stomach of 0.2–6% of humans patients with severe gastric complaints^1^. Infection causes gastritis and peptic ulceration and the relative risk of developing mucosa-associated lymphoid tissue (MALT) lymphoma has been described to be higher with NHPH than with *H. pylori*^1^,^2^. Several studies have shown that *H. suis,* a member of the *H. heilmannii* sensu lato (s.l.) group^3^, is the most prevalent gastric NHPH in humans, which has been described to account for 14% to 78.5% of NHPH infections^4^,^5^,^6^. Interestingly, experimental infection studies in rodent models of human gastric disease have confirmed that long-term *H. suis* infection can lead to the development of gastric MALT lymphoma^7^,^8^,^9^. Besides humans, the majority of pigs worldwide are colonized by this bacterium, in which infection causes chronic gastritis and reduced average daily weight gain^10^. Transmission of *H. suis* most likely occurs through contact between pigs and humans^1^,^11^. Presence of viable *H. suis* bacteria in pork, however, suggests that foodborne infection might also occur^12^.

A persistent mild chronic gastritis can be observed in human patients after *H. heilmannii* s.l. eradication treatment with antibiotics and proton-pump-inhibitors^11^,^13^. For *H. pylori*, it has also been described that after successful eradication in humans, corpus gastritis and dysplasia improve, although they do not completely disappear. Especially antral lesions seem non-responsive after eradication therapy^14^. This emphasizes the need for supplements that promote health of the gastric mucosa.

Recently, we and others identified the γ-glutamyltranspeptidase (GGT) from *H. suis* and *H. pylori* as an important factor causing epithelial cell death and modulating lymphocyte responses^15^,^16^,^17^,^18^. For all gastric helicobacters, the mode of action of the GGT mostly depends on the breakdown of 2 substrates, glutathione (GSH) and glutamine (Gln), both leading to the production of glutamate^8^,^19^,^20^,^21^,^22^. Under certain conditions, GGT-mediated degradation of GSH facilitates the formation of reactive oxygen species, causing lipid peroxidation of cell membranes, which ultimately leads to apoptosis or necrosis of gastric epithelial cells *in vitro*^15^. Depletion of Gln *in vitro* can lead to an impaired proliferation and dysfunction of T-lymphocytes^16^,^17^,^18^.

Gln and GSH have a broad range of functions in the host. Gln plays a crucial role in the energy supply of rapidly dividing cells, such as intestinal epithelial cells and cells of the immune system^23^. In addition, this amino acid plays a role in protein turn-over and purine and pyrimidine synthesis^24^,^25^. The tripeptide GSH (γ-L-glutamyl-L-cysteinylglycine) is synthesized intracellularly. This ubiquitous free thiol is not only important for anti−oxidative protection of the plasma membrane and organelles, but it also plays distinct roles in cell cycle regulation and apoptosis^26^,^27^. After being transported outside the cells, GSH is degraded by eukaryotic membrane-bound GGT which removes the γ-glutamyl moiety^28^. The degradation products can be translocated into the cell, were they can for instance be recycled for GSH synthesis^28^.

Both deamination of Gln and degradation of GSH by *Helicobacter* GGT generate glutamate^15^,^16^,^20^, which can be taken up in the bacterial cell by a Na^+^-dependent transporter^20^,^29^. For *H. pylori*, metabolic reconstruction has suggested that glutamate can subsequently be converted to α-ketoglutarate, which can be channeled into the Tricarboxylic Acid cycle (TCA cycle)^30^. In contrast to most other bacteria, the TCA cycle of *H. pylori* is an atypical, non-cyclic pathway containing both an oxidative and reductive branch^31^. Some genes, necessary for a traditional TCA cycle are missing in the *H. pylori* genome. However, alternative reactions have been identified that allow connectivity between the metabolites of the TCA cycle^30^. No information is currently available on the metabolism of *H. suis*, a gastric *Helicobacter* species which is even far more fastidious than the well-studied *H. pylori*.

The present study is the first to examine the relative importance of the glutamate-generating metabolism (and by extension the amino acid metabolism) for survival and growth of *H. suis.* Since degradation of GSH and Gln by GGT from gastric helicobacters largely contribute to the glutamate-generating metabolism, we investigated the effect of GGT substrate supplementation (Gln/GSH) on the outcome of an experimental *H. suis* infection in Mongolian gerbils. By correcting for possible imbalances, we aimed at reducing infection-related gastric pathologies.

## Results

### The glutamate-generating metabolism is vital for survival and growth of *H. suis*

**Figure 1:**
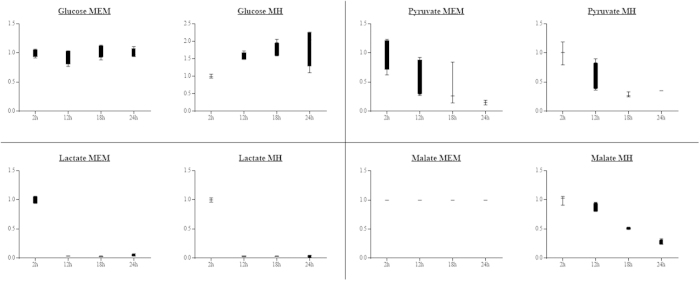
Carbohydrate concentrations in the supernatant of a liquid *Helicobacter suis* culture. Minimal Essential Medium (MEM) and Mueller Hinton (MH) medium. The y-axis represents the fold change of the different carbohydrates relative to the 2 hour measurement point.

Annotation of the *H. suis* genome identified no homologues for some important genes with a putative role in glucose metabolism^21^, suggesting that *H. suis* is unable to utilize the glycolytic pathway for metabolism. The genome of *H. suis* appears to lack glucose permease and several enzymes of the classic Embden-Meyerhof-Parnas pathway, including glucokinase and phosphofructokinase^21^. Furthermore, most components of the Entner-Doudoroff pathway are absent in the *H. suis* genome, such as glucose 1-dehydrogenase and gluconate dehydratase^21^. Utilization of glucose was not observed during *H. suis* culture in both Minimal Essential medium (MEM) and Mueller Hinton (MH) medium (Fig. 1, Table S1), further suggesting this bacterium does not utilize glucose as a major energy source.

**Figure 2:**
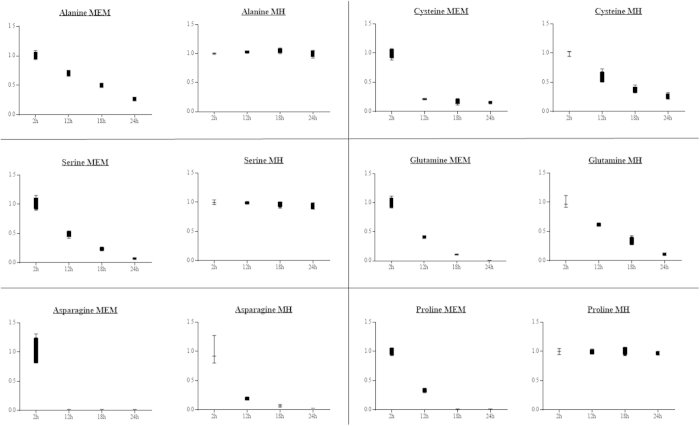
Amino acid concentrations in the supernatant of a liquid *Helicobacter suis* culture. Shown are the relative fold changes of the different amino acid concentrations compared to the 2 hour measurement point. MEM = Minimal Essential Medium, MH = Mueller Hinton.

Instead, we hypothesized that this bacterium has to rely on other sources for its energy production. Pyruvate occupies a central branch point in the energy metabolism of *H. suis*. As the glycolytic pathway is impaired, pyruvate is most likely generated from amino acids such as serine, alanine and cysteine. This hypothesis was confirmed when we found that incubation of *H. suis* for 24 hours in MEM significantly lowered the extracellular concentrations of serine (p < 0.001), alanine (p < 0.001) and cysteine (p < 0.001). A decrease (up to 6-fold) of these amino acids was observed, probably due to the generation of pyruvate. In contrast, in MH-medium only cysteine concentrations significantly decreased (p < 0.001), whereas serine and alanine concentrations remained steady over time (Fig. 2). Genome annotation (using RAST) showed the presence of homologues for *H. pylori* genes encoding for L-serine deaminase (Accession number: NC.000915.1, gen ID 900301), cystathionine γ-synthase (NC.000915.1, gene ID 898970), alanine racemase (NC.000915.1, gen ID 899470) and D-amino acid dehydrogenase (NC.000915.1, gen ID 899472) in the *H. suis* genome (respective locus tags: HSUHS1_RS00280, HSUHS1_RS06050, HSUHS1_RS00415 and HSUHS1_0061)^21^. Another amino acid that was decreased to basal levels after 24 h incubation in MEM was proline (p = 0.001, Fig. 2). We hypothesize that, as described for *H. pylori*, proline is taken up by the bacteria from the extracellular medium—mediated by the proline permease PutP^32^ – and subsequently converted to glutamate inside the bacteria by the proline utilizing flavoenzyme A (PutA)^32^,^33^. A homologue of the *putP* gene of *H. pylori* (NC.00915.1, gen ID 899191) is indeed present in the genome of *H. suis* (locus tag HSUHS1_1153)^21^. No significant changes were detected for this amino acid when *H. suis* was incubated in MH-medium.

Other substrates that decreased over time in the culture supernatant included malate and lactate. In MEM, lactate levels significantly decreased (p < 0.001). In MH-media, lactate, as well as malate concentrations decreased (resp. p = 0.001 and p < 0.001, Fig. 1). They showed up to a 7-fold reduction after *H. suis* culture, suggesting they may also be important carbon sources. A homologue for the *H. pylori* gene encoding for D-lactate dehydrogenase (NC.000915.1, gene ID 898825) is also present in the *H. suis* genome (locus tag HSUHS5_RS01825)^21^, allowing the conversion of lactate to pyruvate.

Pyruvate can subsequently be converted to acetyl-CoA by pyruvate synthase. For *H. pylori,* it has been described that the main route for pyruvate assimilation is via a pyruvate:flavodoxin oxidoreductase (POR) (NC.00915.1, gen ID 899646)^34^. ORF’s have been identified in the *H. suis* genome encoding a POR-type pyruvate synthase (pyruvate oxidoreductase: HSUSH1_RS06160, phosphoenolpyruvate synthase: HSUHS1_RS07385)^21^, suggesting that a similar mechanism is present in *H. suis*. As described for *H. bizzozeronii*, the genome of *H. suis* also codes for phosphoenol pyruvate carboxylase (locus tag HSUHS1_RS00640)^21^, an enzyme that plays a role in the synthesis of oxaloacetate from phosphoenolpyruvate^35^.

Certain amino acids were nearly depleted from the medium of a 24 h culture of *H. suis.* The levels of asparagine decreased approximately 50-fold (p < 0.001, Fig. 2), while the level of Gln dropped below the detection limit (p < 0.001, Fig. 2). Metabolic reconstruction showed that homologs for *HP-ansB/HP-dcuA* and *HP-ggt/HP-gltS* are present in the *H. suis* genome^21^. For *H. pylori*, these hydrolysis/transport systems have been shown to be responsible for the deamination of asparagine and Gln and subsequent uptake by the bacteria of aspartate and glutamate, respectively^29^. These amino acids not only serve as precursors to protein synthesis, they can also be metabolized as energy source via the TCA cycle. Indeed, a homologue for the *H. pylori* gene coding for NADP-dependent glutamate dehydrogenase (NC.000915.1, gen ID 898871) is present in the genome of *H. suis* (locus tag HSUHS1_RS07485)^21^. This enzyme can convert glutamate to 2-ketoglutarate, an intermediate metabolite of the TCA cycle, whereas aspartate can be channeled into the TCA cycle by aspartate ammonia-lyase or other multi-step pathways, such as adenylosuccinate synthase—adenylosuccinate lyase. In any case, this underlines the importance of the amino acid metabolism and Gln metabolism in particular for the growth and energy supply of *H. suis*. A more detailed report of the metabolites used by *H. suis* in both the MEM- and MH-medium can be found in supplementary Table S1.

### Degradation of Gln and GSH by *Helicobacter suis* GGT

**Figure 3:**
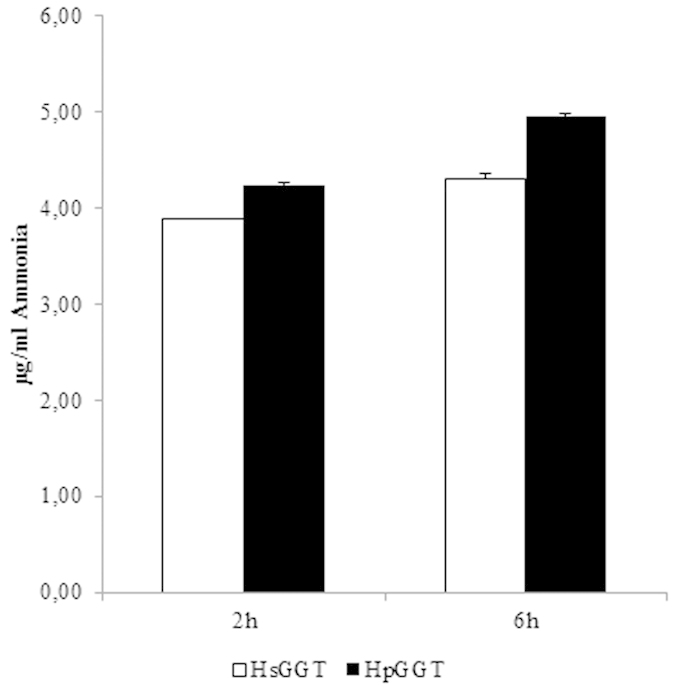
Production of ammonia during *Helicobacter suis*/*H. pylori* GGT-mediated degradation of glutamine.

*H. suis* GGT (HsGGT) and *H. pylori* GGT (HpGGT) were shown to have a very similar Gln- and GSH-degrading capacity. After 2 hours of incubation, 3.88 ± 0.01 μg/ml and 4.23 ± 0.02 μg/ml ammonia was generated by Gln degradation through 2 μg/ml HsGGT and HpGGT, respectively. After 6 hours of incubation, slightly higher amounts of ammonia were generated (4.30 ± 0.06 μg/ml and 4.95 ± 0.02 μg/ml, respectively) (Fig. 3).

**Figure 4:**
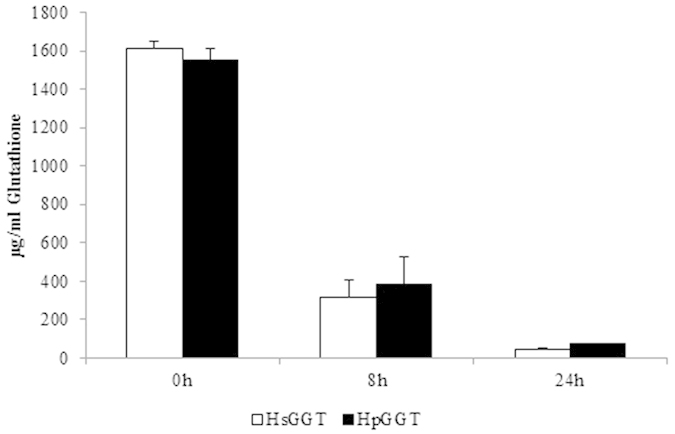
Degradation of GSH by *Helicobacter suis* GGT (HsGGT) and *H. pylori* GGT (HpGGT). After supplementing 2 μg/ml HsGGT or HpGGT to Ham’s F12 containing 1600 µg/ml glutathione (GSH), a decrease in GSH concentration was observed.

After 8 hours of incubation, the initial GSH concentration was reduced by 80%, which added up to 97% after 24 h. Comparable data were obtained for HpGGT. After 8 h, the GSH concentration was reduced by 75%, whereas after 24 h the GSH concentration was reduced by 95% (Fig. 4).

### GSH and Gln supplementation drastically reduces *Helicobacter*-induced inflammation and cell damage

Given the importance of the glutamate-generating metabolism for *H. suis* and the potential impact of the concurrent Gln and GSH degradation (with an important role for *H. suis* GGT in these processes), we investigated the effects of feed supplementation with both substrates to *H. suis* infected Mongolian gerbils.

No differences in feed intake were observed between animals from different groups during the experiment (p > 0.05, Table S2). In addition, animals in different groups showed a similar weight gain during the course of the experiment (Table S2). All uninfected control animals tested negative for the presence of *H. suis*. In experimentally infected animals, the highest *H. suis* colonization rates were found in the antrum. Average colonization rates in animals receiving the standard diet, Gln-supplemented and GSH-supplemented diet were 2.49 × 10^4^ (±2.76 × 10^4^), 1.13 × 10^4^ (±6.08 × 10^4^) and 2.62 × 10^3^ (±5.53 × 10^3^) bacteria/mg tissue, respectively. There were no statistically significant differences between different groups, although animals receiving the GSH diet tended to show lower colonization rates (Standard vs Gln p = 0.606; Standard vs GSH p = 0.129, Fig. S1).

**Figure 5:**
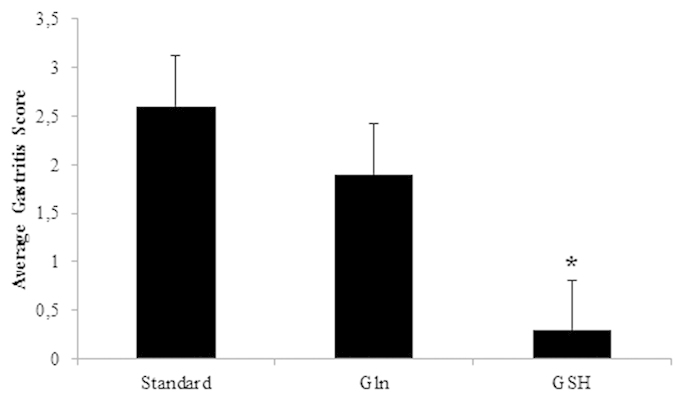
Average gastritis scores observed in the antrum of the stomach of *H. suis* infected animals shows a markedly decreased inflammation in the group receiving the glutathione diet (GSH). An *depicts significantly lower values compared to the *H. suis* infected animals receiving the standard feed. Error bars represent the standard deviation.

**Figure 6:**
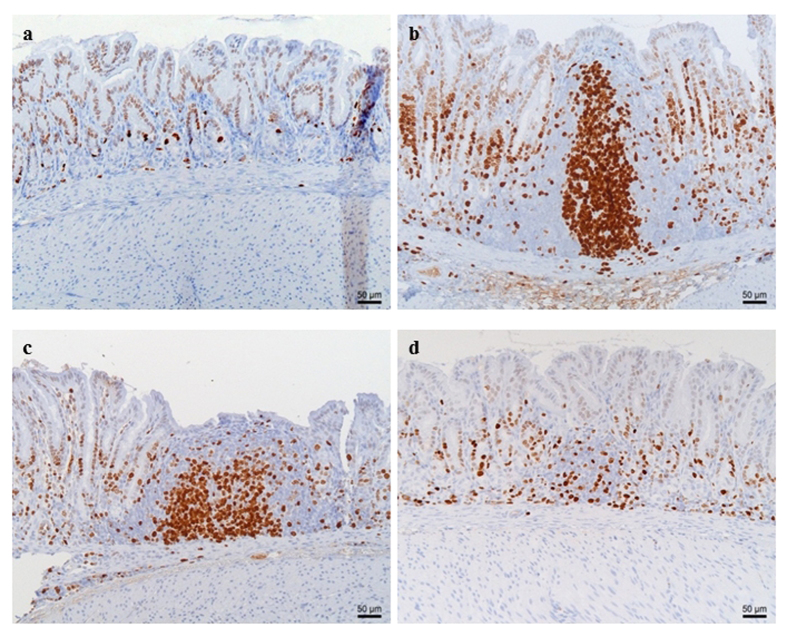
Proliferating lymphocytes in the antrum of control (a)and *Helicobacter suis* infected animals (b–d). Proliferation of lymphocytes, as well as the formation of lymphoid follicles with proliferating germinal centers, is observed in *H. suis* infected animals receiving the standard diet (**b**) and infected animals receiving the glutamine-supplemented diet (**c**). In contrast, only a limited amount of proliferating lymphocytes are observed in *H. suis* infected animals receiving the diet supplemented with glutathione (**d**). In control animals (**a**), only a limited number of cells are proliferating and no lymphocytic aggregates are present. Proliferating Ki-67 positive cells are brown.

None of the control animals showed signs of gastritis. In *H. suis* infected animals, gastritis was mainly present in the antrum. There was a significant difference in gastritis scores between the different groups of infected animals (Fig. 5). A detailed overview of the observed inflammation scores can be found in the online supplementary data (Table S3). Infected animals belonging to the group receiving the GSH supplemented diet showed substantially lower inflammation scores (mean score 0.3 ± 0.4; Fig. 6D) compared to infected animals from the group receiving the purified standard diet (mean score 2.6 ± 0.4; p < 0.001; Fig. 6B). Seven out of 10 *H. suis* infected animals receiving the GSH-supplemented diet even showed no detectable inflammation. Although notably less lymphocytic infiltration and fewer lymphocytic aggregates were also observed in the infected animals receiving the Gln supplemented diet (mean score 1.9 ± 0.9; Fig. 6C), this was not statistically significant (p = 0.064). In general, deep lymphocytic infiltrates consisted mainly of CD3-positive cells whereas superficial lymphoid follicles contained a majority of CD3-negative B lymphocytes (Fig. S2).

**Figure 7:**
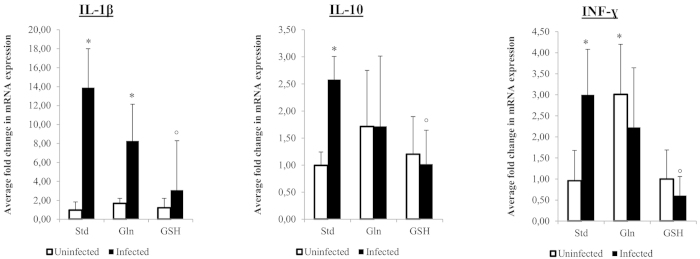
Fold change in cytokine expression in control and *Helicobacter suis* infected animals. An *indicates a significant difference in infected animals compared to the negative control animals receiving the standard diet. An °indicates a significant difference in expression compared to *H. suis* infected animals receiving the standard diet (p < 0.05). Std: animals receiving the standard diet; Gln: Glutamine-supplemented diet; GSH: Glutathione-supplemented diet.

Results of cytokine expression are summarized in Fig. 7. For most cytokines tested, no differences were observed between control groups receiving different diets. Surprisingly, however, uninfected control animals receiving the Gln-supplemented diet did show an upregulation of INF-γ expression (p = 0.012, fold = 3.01 ± 1.19).

When comparing control and *H. suis* infected animals, both receiving the standard diet, *H. suis* infection was shown to provoke an upregulation of IL-1β, INF-γ and IL-10 expression. In *H. suis* infected animals receiving the Gln-supplemented diet, only an upregulation of IL-1β was noted, whereas mRNA expression levels of other cytokines dropped back to the levels of uninfected control animals. Animals receiving the GSH diet even showed cytokine expression levels comparable to those in uninfected control animals.

**Figure 8:**
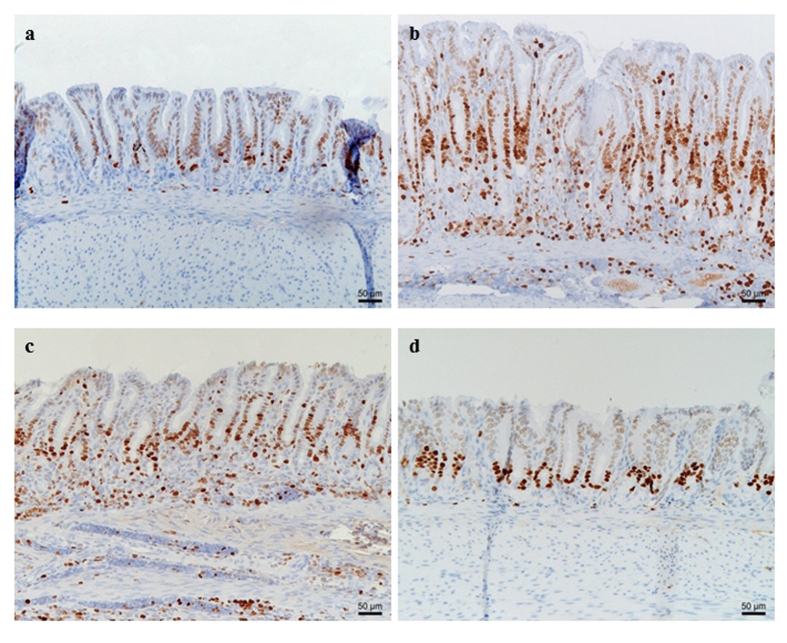
Proliferating Ki67-positive (brown) epithelial cells and lymphocytes in the antrum of the stomach of control and *Helicobacter suis* infected animals. (**a**) Control animal receiving the standard diet; (**b**) *H. suis* infected animal receiving the standard diet; (**c**) *H. suis* infected animal receiving the glutamine-supplemented diet; (**d**) *H. suis* infected animal on the glutathione-supplemented diet. A more pronounced proliferation is observed in *H. suis* infected animals receiving the standard and glutamine-supplemented diet compared to *H. suis* infected animals receiving the glutathione-supplemented diet.

**Figure 9:**
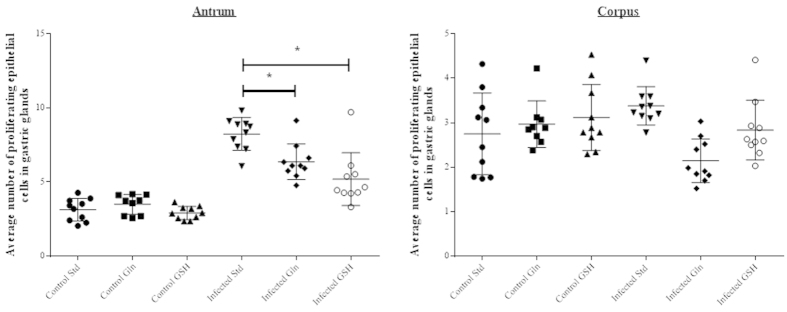
Proliferation of gastric epithelial cells. Ki67 staining of the stomach epithelium in the antrum and corpus of the stomach. Std: standard feed, Gln: glutamine-supplemented feed, GSH: glutathione-supplemented feed. An *depicts significantly lower values compared to the *H. suis* infected animals receiving the standard feed. Error bars represent the standard deviation.

No differences in epithelial cell proliferation were observed between the non-infected groups receiving different diets. Marked differences in terms of gastric epithelial proliferation at the level of the antral pits were observed between *H. suis* infected groups receiving different diets (Figs 8 and 9). *H. suis* infected animals receiving the standard diet showed the highest epithelial proliferation rate (8.2 ± 1.0 Ki67-positive cells per gland; p < 0.001). The proliferation rate was significantly lower in animals receiving the Gln- (6.2 ± 1.1, p = 0.010) or the GSH-supplemented diet (4.9 ± 1.6, p = 0.002) compared to infected animals receiving the non-supplemented diet. In the corpus, no differences in epithelial cell proliferation were observed between the different *H. suis* infected groups (Fig. 9).

Besides their abundant presence in the corpus, a limited number of parietal cells can also be found in the antrum. An abrupt loss of parietal cells was observed in the transition zone between the corpus and antrum in the *H. suis* infected animals receiving the standard diet as well as the Gln-supplemented diet. In *H. suis* infected animals receiving the GSH-supplemented diet, this abrupt loss was absent, revealing an image comparable to that of uninfected control animals (Fig. S3).

## Discussion

Metabolic profiling and the absence of putative genes encoding important enzymes of the Embden-Meyerhof-Parnas and Entner-Doudoroff pathway suggest that, in contrast to the closely related *H. pylori* and *H. bizzozeronii, H. suis* is not able to utilize glucose for its main energy supply. As a consequence, *H. suis* has to rely on other sources to fulfill its energy requirements. Metabolic reconstruction and analysis of metabolites in *H. suis* culture medium showed that *H. suis* tends to use amino acids, not only for protein synthesis, but most likely also as an energy source. The consumption of amino acids was obvious in cultures grown in both liquid media, but was most pronounced in MEM, which can be considered to be the most basic one of the two. This could indicate that *H. suis* relies even more on amino acids for its survival when facing stressful situations.

One of the preferred nutrients seems Gln, which is the most abundant free amino acid in both plants and animals^36^,^37^,^38^. Our current and previous studies have shown that Gln is depleted from the medium during *H. suis* culture due to the hydrolysis by *H. suis* GGT^16^. As described for *H. pylori,* the formed glutamate can be taken up and converted to 2-ketoglutarate by the bacteria and channeled into the TCA cycle^21^,^29^, an important metabolic pathway that provides precursors for a variety of cell components, as well as for supplying energy to the bacterium^31^.

The hydrolysis of extracellular Gln by the GGT from gastric helicobacters can cause a depletion of this conditionally essential amino acid in the gastric juice of an infected host, which may eventually lead to epithelial cell damage and immunological dysfunction^16^,^18^,^20^. By supplementing Gln to the diet of Mongolian gerbils we aimed at reducing inflammation-related as well as other *Helicobacter-*related gastric pathological changes. In addition to a local effect, dietary Gln supplementation has been described to cause an increase of the systemic Gln concentration, which may also sustain the health and function of immune and epithelial cells^39^. The role of Gln in gastric mucosal protection against *H. pylori* infection has been reported previously^36^,^37^,^38^,^40^,^41^,^42^. In these studies, an effect of Gln supplementation on gastric inflammation and cytokine expression was observed^41^. In addition, a recent study indicated that the depletion of Gln, as a result of high GGT activity, might actually be responsible for the strong inflammatory response following *H. pylori* infection, as shown by increased release of pro-inflammatory IL-8 from epithelial cells^17^. In contrast, Wüstner *et al*.^18^ described that *H. pylori* GGT activity leads to an impaired T-cell function by depriving activated lymphocytes from Gln.

In the present study, the beneficial effect of Gln supplementation on inflammation was only mild to moderate, which may be due to the shorter infection period compared to that described by Hagen *et al*.^41^. Nevertheless, *H. suis* infected animals receiving the Gln diet showed smaller gastric lymphoid follicles and less diffuse lymphoid infiltration compared to the animals receiving the standard diet. This decreased proliferation of lymphocyte populations, as well as the reduced expression of pro- and anti-inflammatory cytokines in *H. suis* infected animals receiving Gln-supplemented feed is somewhat in contrast to *in vitro* studies showing that Gln supplementation restores the impaired proliferation of *H. suis* GGT-treated Jurkat T-cells^16^. Other factors are most likely involved, such as a protective effect of Gln on the epithelial barrier.

Indeed, the current study is the first to show a direct effect of Gln supplementation on the proliferation rate of gastric epithelial cells in the context of a *Helicobacter* infection, suggesting a protective effect of Gln on gastro-intestinal epithelial cells^43^,^44^,^45^. Most likely, hyperproliferation of epithelial cells found in *H. suis* infected animals receiving the standard diet is a secondary response to increased cell death induced by this bacterium^8^,^45^,^46^. Increased cell death may be induced by an increased oxidative stress burden or a modification of the redox balance of gastric epithelial cells following Gln depletion^15^,^47^. Supplementing Gln may therefore assist in restoring the redox balance. Besides oxidative stress, *H. suis* infection causes a dramatic increase of ammonia concentrations, resulting from urease and GGT activity of the bacterium^1^,^20^,^42^,^48^. Adding Gln to the diet can stimulate arginase activity, causing a conversion of ammonia to the less toxic urea, and helping in clearing ammonia from the cells by stimulating its excretion^23^,^42^. Although it apparently has a beneficial effect on compensatory hyperproliferation of gastric epithelial cells, supplementing Gln to *H. suis* infected animals does not prevent the abrupt loss of parietal cells at the transition zone between the corpus and antrum^8^.

Besides a depletion of Gln, GGT from gastric helicobacters also causes degradation of GSH^15^,^16^. Previous *in vitro* research has revealed that supplementation of GSH at a concentration of 5 mM to *H. suis* GGT-treated epithelial cells can stimulate the formation of pro-oxidative GSH degradation products and extracellular oxygen radicals, leading to necrosis of damaged cells^15^. In the present study, however, supplementing 0.8% GSH to the diet of *H. suis* infected gerbils revealed tremendously beneficial effects on the health of a *Helicobacter*-infected stomach. We observed a marked decrease of gastric epithelial cell proliferation rates, protection against parietal cell death and a 90% reduction of gastritis, accompanied by normalized expression levels for all (pro-inflammatory) cytokines, confirming the suppression of the host immune response elicited by *Helicobacter* infection. Only three animals had small lymphoid follicles in the antrum of the stomach, despite normal *H. suis* colonization rates. *Helicobacter* GGT has been shown to inhibit proliferation of T-lymphocytes, an effect that has been suggested to play a role in the immune escape by these bacteria^16^,^49^. Supplementing GSH to the cell medium has been shown to aggravate this effect^16^. Possibly, similar mechanisms are involved in the current *in vivo* study.

The observed protective effects on infection-induced inflammation and, surprisingly, epithelial cell hyperproliferation may be explained by a dose-dependent effect of GSH. Intracellular concentrations of GSH are usually very high, ranging from 0.5 to 10 mM, whereas extracellular concentrations are found in the micromolar range^50^. Therefore, supplementing feed with higher concentration of GSH (35 mM) compared to the dosages used for previous *in vitro* studies (5 mM)^15^ probably results in an excess quantity of protective extracellular GSH, most likely counterbalancing some potentially negative effects on gastric epithelial cell health by GGT-induced generation of pro-oxidative GSH degradation products^15^. It has indeed been shown that, when available for the host cells, GSH plays an important role in the protection of cells against oxygen radicals, detoxification and metabolisation of various endo- and exogenous compounds, cell cycle regulation, cell signaling and apoptosis^16^,^50^,^51^,^52^. GSH deficiency makes cells more sensitive to oxidative stress and can cause degeneration of epithelial cells^50^,^51^. The remarkable beneficial effects of GSH on *Helicobacter*-induced inflammation and epithelial cell homeostasis suggest that GSH supplementation could be considered as a supportive therapy during and even after a *Helicobacter* eradication therapy. It should be noted, however, that only the short/medium-term effects of GSH supplementation were evaluated during this trial. A study of Liu *et al*.^53^ showed that GSH supplementation had beneficial effects on acute clinical signs of inflammatory bowel disease (IBD), whereas some adverse effects were observed in the chronic stages of IBD in this particular disease model. More research is therefore needed to evaluate the long-term safety and effects of GSH supplementation and to make recommendations on the chronic use of glutathione in people suffering from gastric disease.

Feed additives are commonly used in the pig industry to promote pig health. They are easily manufactured and provided to the animals. Both Gln and GSH can be considered as a possible feed additive since the concentrations of both were shown to be stable in the feed pellets used in the present study (unpublished results). These supplements may therefore be helpful for decreasing both the prevalence and severity of gastric pathologies caused by *H. suis* infection in pigs. Indeed, up to 90% of *H. suis* infected pigs have been shown to develop gastritis, which most likely plays a role in the observed reduction of daily weight gain, a primordial production characteristic in the pig industry^10^.

In conclusion, we showed that *H. suis* preferably uses amino acids to meet its energy requirements. In particular, the glutamate-generating metabolism seems essential, as shown by a complete depletion of Gln during *H. suis* culture. The *H. suis* GGT has previously been shown to be an important factor degrading Gln, but also GSH. Oral supplementation of *H. suis* infected animals with these two substrates, Gln or GSH, resulted in a marked reduction of infection-related gastric pathologies, both related to inflammation and epithelial cell damage. GSH was shown to be superior to glutamine and may prove to be of therapeutic value for use in both humans and animals.

## Methods

### Metabolic Profiling of *H. suis*: Energy Metabolism

*H. suis* type strain HS1LP was grown in liquid Minimal Essential Medium (MEM) and Mueller Hinton (MH) broth. The sample preparation process was carried out using the automated MicroLab STAR^®^ system (Hamilton Company, Reno, US). Recovery standards were added prior to the first step in the extraction process for Quality Control purposes. Sample preparation was conducted using a proprietary (Metabolon, Durham, North Carolina, USA) series of organic and aqueous extractions to remove the protein fraction while allowing maximum recovery of small molecules. The resulting extract was divided into two fractions, one for analysis by Liquid Chromatography (LC) and one for analysis by gas chromatography (GC). Samples were placed briefly on a TuboVap^®^ (Zymark, Massachusetts, US) to remove the organic solvent. Each sample was then frozen and dried under vacuum. Samples were then prepared for the appropriate instrument, either LC/MS or GC/MS.

The LC/MS portion of the platform was based on a Surveyor HPLC and a Thermo-Finnigan LTQ Fmass spectrometer (Thermo scientific, Waltham, Massachusettes, USA), which consisted of an electrospray ionization (ESI) source and linear ion-trap (LIT) mass analyzer. Positive and negative ions were monitored within a single analysis by consecutively alternating the ionization polarity of adjacent scans. The vacuum-dried sample was dissolved in 100.0 μl of an injection solvent that contained five or more injection standards at fixed concentrations. The chromatography has been standardized by Metabolon, Inc. and once a method was validated, no changes were made to it. The internal standards were used both to assure injection and chromatographic consistency. The chromatographic system used a binary solvent (water and methanol) system delivered as a gradient. The HPLC columns were washed and reconditioned after every injection.

The samples destined for GC/MS analysis were re-dried under vacuum desiccation for a minimum of 24 hours prior to being derivatized under dried nitrogen using bistrimethyl-silyl-triflouroacetamide (BSTFA). The GC column was 5% phenyl and the temperature ramp ranged from 40° to 300 °C in a 16 minute period. Samples were analyzed on a Thermo-Finnigan Trace DSQ fast-scanning single-quadrupole mass spectrometer using electron impact ionization.

At 2, 12, 18 and 24 hours media supernatants was analyzed. For each condition, 4 biological replicates were performed. A small aliquot of each experimental sample for a specific matrix was obtained and pooled. These pooled samples were injected throughout the platform day run and served as technical replicates.

In total, 702 media were identified and statistical analysis was performed to detect concentration changes during *H. suis* culture. Based on information derived from a draft genomic sequence^21^, a partial metabolic reconstruction of *H. suis* strains HS1 and HS5 (currently named *H. suis* strains HS1LP and HS5bLP^54^) was performed, mainly focusing on the carbon metabolism of this bacterium and attempting to confirm the results of metabolic profiling (Integrated Genomics Inc., Chicago, Illinois, US).

In addition, the RAST (Rapid Annotation using Subsystem Technology) tool was used to confirm the presence/absence of genes important for the metabolism of *H. suis* in the genome of *H. suis* strains HS1LP and HS5bLP^21^,^54^.

### Effects of *H. suis* and *H. pylori* GGT on Gln and GSH

The *H. suis* and *H. pylori* GGT were expressed and purified as described previously^15^. Both enzymes were compared to each other regarding their capacity to degrade 2 important substrates: Gln and GSH. Two μg/ml of *H. suis* or *H. pylori* GGT was added to HBSS supplemented with 2 mM Gln and incubated at 37 °C for 2 hours and 6 hours. As a control, a solution of HBSS supplemented with 2 mM Gln was used. The concentration of produced ammonia was detected using an Ammonia Assay Kit (Abcam, Cambridge, UK) according to the manufacturer’s instructions.

To evaluate the effect of HsGGT and HpGGT on GSH, 1600 µg/ml GSH was incubated for 8–24 hours at 37 °C with 2 μg/ml HsGGT or HpGGT in Ham’s F12 medium. GSH concentration was measured using the Glutathione Assay Kit (Sigma-Aldrich) according to the manufacturer’s instructions.

### Bacterial strain used for *in vivo* experimentation

*H. suis* strain HS5bLP, isolated from the gastric mucosa of a sow^55^, was used for experimental infection of Mongolian gerbils. Culture was performed as described previously^54^.

### Ethics statement

All experimental procedures were approved by the Ethical Committee of the Faculty of Veterinary Medicine of the University of Ghent, Belgium (approval number EC2011-113), and carried out in accordance with the approved guidelines and regulations.

### Animals, housing, inoculation and sampling

Sixty female Mongolian gerbils (Charles River, France) arrived at our animal housing units at the age of 4 weeks. They were randomly divided in 6 groups with 10 animals each. Animals from 1 group were divided in 2 filter-top cages (5 animals each), all held in the same stable under controlled environmental conditions. During the experiment, feed intake was monitored weekly and body weight was measured three times, twice at inoculation and once at euthanasia.

Three different rodent diets (Table 1) were provided ad libitum to 20 animals each (Research Diets, Inc., New Brunswick, USA). The standard diet was a purified feed (AIN-93 G, Research Diets, Inc.). The other 2 diets were identical to the standard diet, with the exception of being supplemented with 5% Gln or 0.8% GSH and with a variable amount of corn starch, used to correct minor differences in the energy content. At the age of 6 weeks, the animals were briefly anaesthetized with 3% isoflurane and inoculated intragastrically twice, with a two-day interval. Ten animals from each feed group received 8 × 10^7^ viable *H. suis* bacteria/dose. The remaining 10 animals/feed group were sham-inoculated with the culture medium of *H. suis*.

Twelve weeks after the first inoculation, animals were anaesthetized with 5% (vol/vol) isoflurane followed by cervical dislocation. The stomach was opened along the greater curvature. A longitudinal strip was taken from the forestomach to the duodenum, fixed in 4% phosphate buffered formaldehyde and embedded in paraffin for histopathological examination. Biopsies from antrum and corpus were taken for DNA and RNA extraction. These tissues were immediately submerged in RNA*later* (Qiagen, Hilden, Germany) and stored at −70 °C until further processing.

### DNA extraction and quantitative-PCR to determine the colonization rate of *H. suis*

DNA extraction and Quantitative-PCR were performed on the DNA samples as described previously^56^,^57^.

### RNA extraction and Real-Time PCR analysis of cytokine expression

After separation of DNA and RNA, the latter was further purified using the RNeasy Mini kit (Qiagen) following the suppliers’ instructions. Synthesis of cDNA was done by using the iScript^TM^ cDNA synthesis kit (Bio-rad, Hercules, USA) as described by the manufacturer. Real Time−PCR and melting curve analysis were done as described before^58^. The primer sequences are summarized in Table 2. Primers for interleukin (IL)-5 were designed using primer3 software.

### Histopathology

Four sections of 5 μm were sliced from the paraffin-embedded stomach tissue. The first section was used for a hematoxylin and eosin (HE) staining to score the intensity of infiltration with mononuclear and polymorphonuclear cells. A second tissue slide was stained using a mouse monoclonal anti-Ki67 antibody (Menarini Diagnostics, Firenze, Italy; dilution 1:50) and a biotinylated goat anti-mouse IgG antibody to identify proliferating cells. Positive cells lining the lumen of the gastric pits were counted in five randomly chosen High Power Fields (magnification: x400) in the corpus and antrum. For each animal, an average of the positive cell count was determined for both stomach regions. A third section was used to stain acid-producing parietal cells of the corpus of the stomach by using an antibody directed against the H^+^K^+^ATP−ase β-subunit (Abcam, Cambridge, UK; dilution 1:25000). The fourth tissue slide was used for visualization of T-lymphocytes, by using an antibody directed against CD3 (Abcam, dilution 1:100).

### Statistical analysis

T-tests were used to analyze the data of the metabolomics study. T-test comparisons were performed between each experimental group and its control.

Differences in gastritis between experimentally infected animals and control animals receiving the standard diet were analyzed using a Mann-Whitney U non-parametric test. Comparison of the colonization rates, cytokine expression and epithelial proliferation rates between the different diet groups of infected animals was done by means of a one-way ANOVA (SPSS 21, IBM, New York, USA). P-values ≤ 0.05 were considered statistically significant. When multiple groups were compared, a Bonferroni correction was made. All data are expressed as mean ± standard deviation.

## Additional Information

**How to cite this article**: De Bruyne, E. *et al*. Oral glutathione supplementation drastically reduces *Helicobacter*-induced gastric pathologies. *Sci. Rep.* **6**, 20169; doi: 10.1038/srep20169 (2016).

## Supplementary Material

Supplementary Information

## Figures and Tables

**Table 1:** Composition of the 3 different diets used in this study.

	**Standard diet**		**Gln-supplemented diet**		**GSH-supplemented diet**	
	**Gram%**	**Kcal%**	**Gram%**	**Kcal%**	**Gram%**	**Kcal%**
Protein	20	20	25	25	20	20
Carbohydrate	64	64	59	59	63	64
Fat	7	16	7	16	7	16
	Gram	Kcal	Gram	Kcal	Gram	Kcal
Casein	200	800	200	800	200	800
L-Cystine	3	12	3	12	3	12
Supplemented L-Glutamine	0	0	50	200	0	0
Corn Starch	397.486	1590	374.486	1390	397.486	1590
Maltodextrin	132	528	132	528	132	528
Sucrose	100	400	100	400	100	400
Cellulose	50	0	50	0	50	0
Soybean oil	70	360	70	360	70	360
Mineral Mix	35	0	35	0	35	0
Vitamin Mix	10	40	10	40	10	40
Choline Bitartrate	2.5	0	2.5	0	2.5	0
Supplemented Glutathione	0	0	0	0	8.07	0
Total	1000	4000	1000.05	4000	1008.12	4000

**Table 2:** Forward (F) and reverse (R) primers used for cytokine-expression.

**Cytokine**	**Primers Sequence (5′ → 3′)**	**Reference**
IL-17	F: AGC TCC AGA GGC CCT CGG AC	59
	R: AGG ACC AGG ATC TCT TGC TG	
TNF-α	F: GCT CCC CCA GAA GTC GGC G	59
	R: CTT GGT GGT TGG GTA CGA CA	
IL-10	F: GGT TGC CAA GCC TTA TCA GA	56
	R: GCT GCA TTC TGA GGG TCT TC	
IL-1β	F: GGC AGG TGG TAT CGC TCA TC	Modified from^59^,
	R: CAC CTT GGA TTT GAC TTC TA	
IL-5	F: AGA GAA GTG TGG CGA GGA GAG ACG	Present study
	R: ACA GGG CAA TCC CTT CAT CGG	
IL-6	F: CAA AGC CAG AGC CAT TCA GAG	56
	R: GCC ATT CCG TCT GTG ACT CCA GTT TCT CC	
IL-12p40	F: GAC ACG ACC TCC ACC AAA GT	56
	R: CAT TCT GGG ACT GGA CCC TA	
IFN-γ	F: CCA TGA ACG CTA CAC ACT GCA TC	60
	R: GAA GTA GAA AGA GAC AAT CTG G	
Β-actin	F: TCC TCC CTG GAG AGG AGC TA	61
	R: CCA GAC AGC ACT GTG TTG GC	
HPRT	F: CTC ATG GAC TGA TTA TGG ACA G	61
	R: AGC TGA GAG ATC ATC TCC ACC AAT	
