# Supplementary material for: Oral glutathione supplementation drastically reduces Helicobacter-induced gastric pathologies
Source: Sci Rep. 2016 Feb 2;6:20169. doi: 10.1038/srep20169 (PMC4735851; doi:10.1038/srep20169)
Supplement: Supplementary Information [file srep20169-s1.pdf]

# ORAL GLUTATHIONE SUPPLEMENTATION DRASTICALLY REDUCES *HELICOBACTER*-INDUCED GASTRIC PATHOLOGIES

Ellen De Bruyne, Richard Ducatelle, Dennis Foss, Margaret Sanchez, Myrthe Joosten, Guangzhi Zhang, Annemieke Smet, Frank Pasmans, Freddy Haesebrouck, Bram Flahou

**Figure S1:** *Helicobacter suis* colonization in the antrum of experimentally infected Mongolian gerbils receiving a standard diet (Std), glutamine-supplemented diet (Gln) or glutathione supplemented diet (GSH).

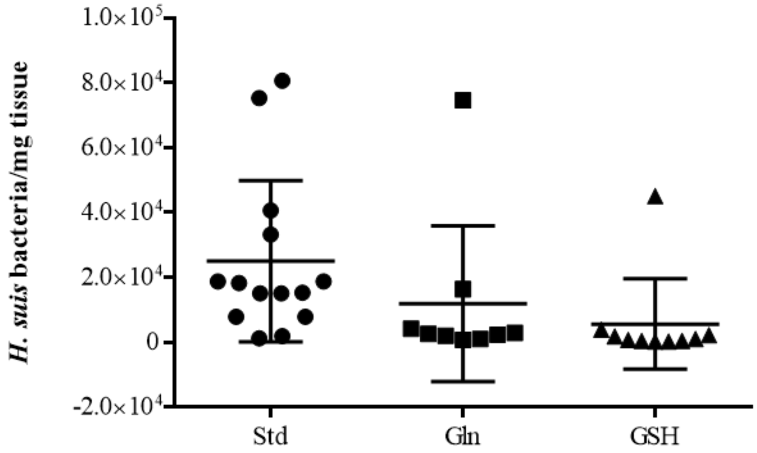

**FIGURE S2:** CD3 staining of the antrum of a control animal (A) and a *Helicobacter suis* infected animal (B), both receiving the standard diet. CD3 positive T-cells (brown) are only present in low numbers in the antrum of control animals receiving the standard diet (A). In contrast, they are abundantly present in the antrum of *Helicobacter suis* infected animals receiving the standard diet (B). Superficial lymphoid aggregates (*) contain more CD3 negative T-cells, whereas deep lymphoid aggregates (°) are mainly composed of CD3 positive cells.

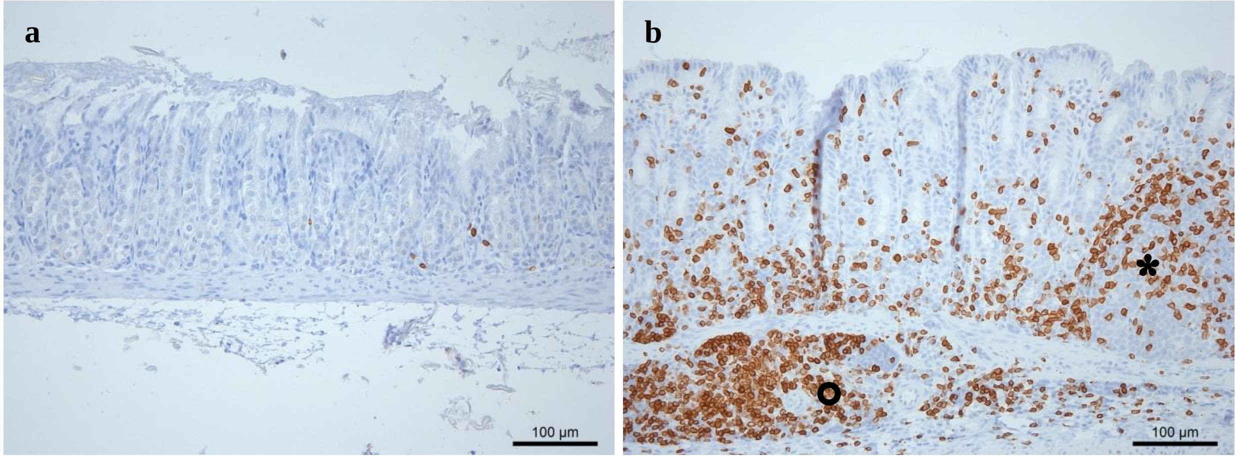

**FIGURE S3:** Immunohistochemical staining of the parietal cell hydrogen potassium ATPase. $H^+$, $K^+$ ATPase-positive parietal cells (brown) in the antrum/antrum-corpus transition zone. A: control animals receiving the standard diet; B: *H. suis* infected animal with the standard diet; C: *H. suis* infected animal receiving the glutamine-supplemented diet; D: *H. suis* infected animal receiving the glutathione-supplemented diet. An abrupt loss of parietal cells is observed in *H. suis* infected animals receiving the standard diet and *H. suis* infected animals receiving the Gln-supplemented diet, but not in *H. suis* infected animals receiving the GSH-supplemented diet.

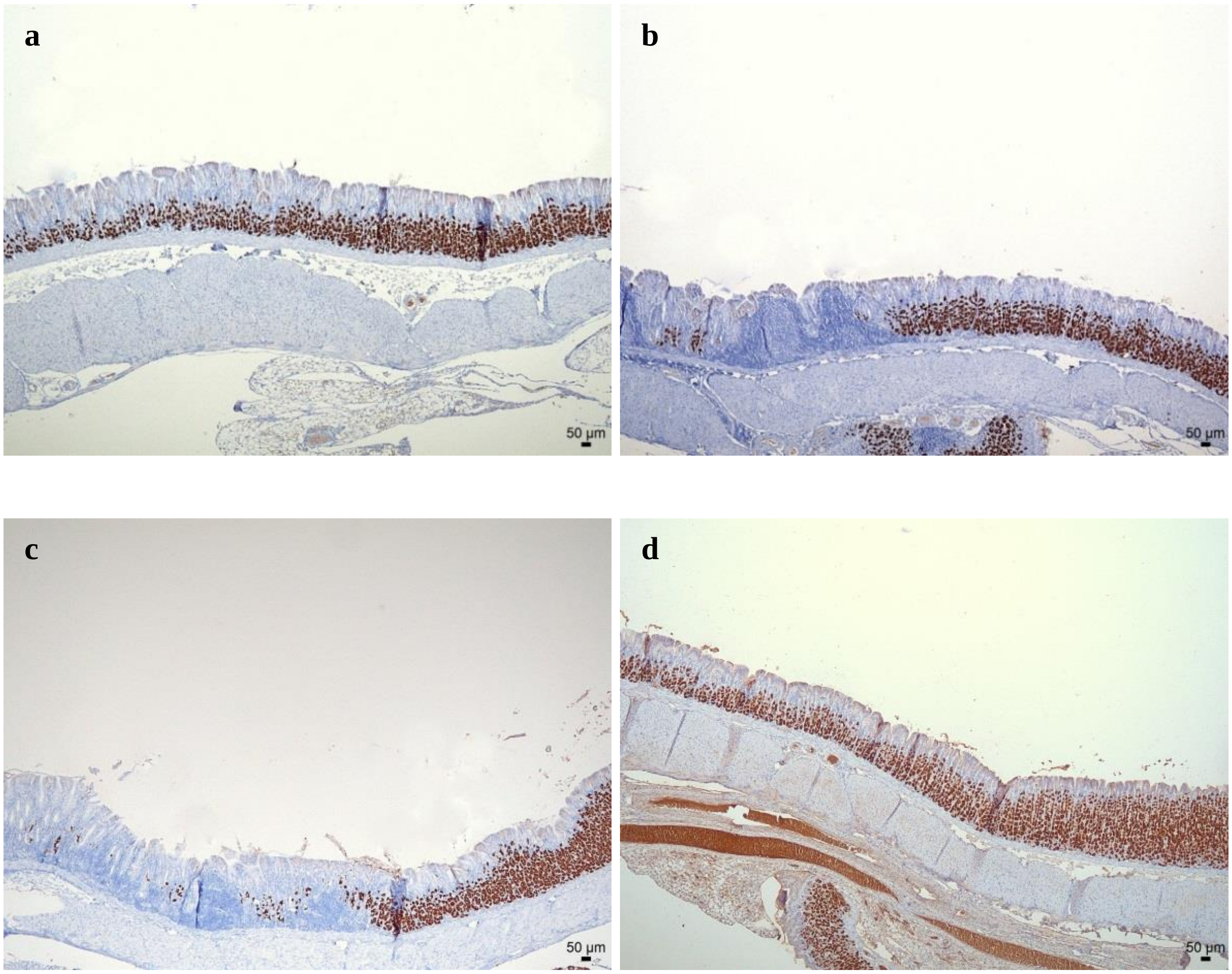

**Table S1:** The fold change of the metabolites that were significantly different in Minimal Essential Medium (MEM) and/or Mueller Hinton Medium (MH) after 12, 18 and 24 hours of incubation with *Helicobacter suis* compared to the 2 hour incubation point. Highlighted in green are the metabolites that significantly decreased over time, highlighted in red are the values that significantly increased. The p-value is given of each measurement.

| | **MEM** | | | | | | **MH** | | | | | |
|---|---|---|---|---|---|---|---|---|---|---|---|---|
| **Name** | **Hour 12 / Hour 2** | | **Hour 18 / Hour 2** | | **Hour 24 / Hour 2** | | **Hour 12 / Hour 2** | | **Hour 18 / Hour 2** | | **Hour 24 / Hour 2** | |
| | **Fold Change** | **P-value** | **Fold Change** | **P-value** | **Fold Change** | **P-value** | **Fold Change** | **P-value** | **Fold Change** | **P-value** | **Fold Change** | **P-value** |
| **Serine** | 0.50 | 0.00 | 0.24 | 0.00 | 0.07 | 0.00 | 1.01 | 0.74 | 0.99 | 0.84 | 0.95 | 0.27 |
| **Betaine** | 1.11 | 0.25 | 1.20 | 0.04 | 1.28 | 0.02 | 0.99 | 0.48 | 0.98 | 0.47 | 1.01 | 0.75 |
| **Alanine** | 0.71 | 0.00 | 0.49 | 0.00 | 0.27 | 0.00 | 1.03 | 0.09 | 1.05 | 0.09 | 1.00 | 0.97 |
| **Beta-alanine** | 0.85 | 0.46 | 0.99 | 0.80 | 1.18 | 0.39 | 1.05 | 0.64 | 0.97 | 0.95 | 1.11 | 0.60 |
| **Aspartate** | 0.36 | 0.00 | 0.19 | 0.00 | 0.12 | 0.00 | 0.45 | 0.00 | 0.23 | 0.00 | 0.13 | 0.00 |
| **Asparagine** | 0.02 | 0.00 | 0.02 | 0.00 | 0.02 | 0.00 | 0.20 | 0.00 | 0.05 | 0.00 | 0.03 | 0.00 |
| **Glutamate** | 2.59 | 0.00 | 3.60 | 0.00 | 3.35 | 0.00 | 1.04 | 0.27 | 1.06 | 0.18 | 1.05 | 0.30 |
| **Glutamine** | 0.41 | 0.00 | 0.11 | 0.00 | 0.00 | 0.00 | 0.64 | 0.00 | 0.34 | 0.00 | 0.12 | 0.00 |
| **3-methyl-2-oxovalerate** | 3.94 | 0.00 | 5.23 | 0.00 | 5.75 | 0.00 | 1.92 | 0.00 | 2.34 | 0.00 | 2.51 | 0.00 |
| **Isoleucine** | 0.92 | 0.06 | 0.90 | 0.03 | 0.92 | 0.01 | 1.05 | 0.19 | 1.04 | 0.42 | 1.01 | 0.80 |
| **Leucine** | 0.91 | 0.02 | 0.89 | 0.02 | 0.90 | 0.01 | 1.06 | 0.10 | 1.06 | 0.17 | 1.02 | 0.62 |
| **Valine** | 0.97 | 0.46 | 0.89 | 0.08 | 0.89 | 0.04 | 1.06 | 0.14 | 1.06 | 0.09 | 1.04 | 0.32 |
| **Cysteine** | 0.21 | 0.00 | 0.18 | 0.00 | 0.16 | 0.00 | 0.57 | 0.01 | 0.36 | 0.00 | 0.26 | 0.00 |
| **N-acetylcysteine** | 0.82 | 0.10 | 0.71 | 0.02 | 0.97 | 0.81 | 0.71 | 0.18 | 0.44 | 0.00 | 0.47 | 0.00 |
| **Cystine** | 1.76 | 0.11 | 2.88 | 0.00 | 2.87 | 0.00 | 2.04 | 0.01 | 2.61 | 0.00 | 2.82 | 0.00 |
| **Methionine** | 0.88 | 0.02 | 0.83 | 0.01 | 0.81 | 0.00 | 1.08 | 0.08 | 1.07 | 0.12 | 1.01 | 0.77 |
| **Ornithine** | 0.87 | 0.11 | 0.81 | 0.01 | 0.91 | 0.21 | 1.00 | 0.96 | 0.98 | 0.84 | 1.06 | 0.46 |
| **Proline** | 0.34 | 0.00 | 0.01 | 0.00 | 0.01 | 0.00 | 0.99 | 0.86 | 1.02 | 0.74 | 0.97 | 0.28 |
| **N-acetylornithine** | 0.90 | 0.57 | 1.11 | 0.48 | 0.98 | 0.79 | 1.00 | | 1.00 | | 1.00 | |
| **Creatine** | 1.03 | 0.52 | 1.24 | 0.00 | 1.29 | 0.04 | 0.92 | 0.26 | 0.94 | 0.35 | 0.94 | 0.38 |
| **Glutamylglutamate** | 1.00 | | 1.00 | | 1.00 | | 0.96 | 0.53 | 1.00 | 0.98 | 0.80 | 0.01 |
| **Prolylleucine** | 1.00 | | 1.00 | | 1.00 | | 1.01 | 0.58 | 1.00 | 0.91 | 0.95 | 0.04 |

| | | | | | | | | | | | | |
|---|---|---|---|---|---|---|---|---|---|---|---|---|
| **Leucylproline** | 1.00 | | 1.00 | | 1.00 | | 0.53 | 0.10 | 1.07 | 0.72 | 0.41 | 0.02 |
| **Pro-hydroxy-pro** | 0.91 | 0.26 | 0.89 | 0.12 | 0.72 | 0.02 | 1.00 | | 1.00 | | 1.00 | |
| **Threonylphenylalanine** | 1.00 | | 1.00 | | 1.00 | | 1.13 | 0.01 | 1.07 | 0.04 | 1.04 | 0.54 |
| **Carnosine** | 1.10 | 0.34 | 1.10 | 0.21 | 1.14 | 0.06 | 1.26 | 0.03 | 1.20 | 0.19 | 1.34 | 0.03 |
| **Fructose** | 0.99 | 0.83 | 1.05 | 0.44 | 1.09 | 0.02 | 1.03 | 0.32 | 1.09 | 0.01 | 1.01 | 1.00 |
| **Maltose** | 1.00 | | 1.00 | | 1.00 | | 1.78 | 0.00 | 1.83 | 0.00 | 1.89 | 0.02 |
| **Sorbitol** | 0.70 | 0.04 | 0.99 | 0.96 | 1.03 | 0.73 | 1.45 | 0.13 | 1.16 | 0.41 | 0.98 | 0.79 |
| **Glycerate** | 1.13 | 0.00 | 1.21 | 0.04 | 1.35 | 0.03 | 1.15 | 0.12 | 1.27 | 0.02 | 1.20 | 0.06 |
| **Glucose** | 0.94 | 0.38 | 1.04 | 0.60 | 1.00 | 0.96 | 1.58 | 0.00 | 1.75 | 0.00 | 1.89 | 0.04 |
| **Pyruvate** | 0.58 | 0.12 | 0.34 | 0.05 | 0.14 | 0.00 | 0.59 | 0.05 | 0.27 | 0.00 | 0.26 | 0.00 |
| **Lactate** | 0.04 | 0.00 | 0.03 | 0.00 | 0.05 | 0.00 | 0.03 | 0.00 | 0.03 | 0.00 | 0.04 | 0.00 |
| **Arabitol** | 0.78 | 0.13 | 0.87 | 0.33 | 0.97 | 0.84 | 1.27 | 0.05 | 1.07 | 0.43 | 1.01 | 0.92 |
| **Ribitol** | 0.37 | 0.00 | 0.41 | 0.01 | 0.35 | 0.00 | 0.84 | 0.38 | 0.67 | 0.06 | 0.55 | 0.05 |
| **Ribose** | 0.69 | 0.02 | 0.86 | 0.37 | 1.03 | 0.87 | 1.00 | | 1.00 | | 1.00 | |
| **Xylonate** | 2.62 | 0.00 | 3.37 | 0.00 | 3.94 | 0.00 | 1.23 | 0.09 | 1.18 | 0.17 | 1.18 | 0.17 |
| **Citrate** | 1.20 | 0.22 | 1.44 | 0.10 | 1.50 | 0.03 | 1.13 | 0.03 | 1.11 | 0.01 | 1.01 | 0.89 |
| **Succinate** | 0.12 | 0.00 | 0.10 | 0.00 | 0.08 | 0.00 | 3.53 | 0.00 | 3.50 | 0.00 | 2.49 | 0.00 |
| **Fumarate** | 1.00 | | 1.00 | | 1.00 | | 1.03 | 0.62 | 0.81 | 0.18 | 0.61 | 0.00 |
| **Malate** | 1.00 | | 1.00 | | 1.00 | | 0.89 | 0.09 | 0.53 | 0.00 | 0.27 | 0.00 |
| **Oxaloacetate** | 1.01 | 0.83 | 1.08 | 0.47 | 0.80 | 0.76 | 1.81 | 0.20 | 1.01 | 0.99 | 1.53 | 0.27 |
| **Phosphate** | 0.37 | 0.00 | 0.23 | 0.00 | 0.16 | 0.00 | 0.97 | 0.28 | 0.93 | 0.04 | 0.92 | 0.02 |
| **Dihomo-linolenate** | 1.93 | 0.00 | 2.49 | 0.00 | 2.92 | 0.00 | 1.37 | 0.03 | 1.58 | 0.07 | 1.84 | 0.00 |
| **Docosapentaenoate** | 2.05 | 0.04 | 2.16 | 0.04 | 2.87 | 0.01 | 1.26 | 0.05 | 1.47 | 0.11 | 1.64 | 0.02 |
| **Docosahexaenoate** | 1.69 | 0.02 | 1.83 | 0.01 | 2.20 | 0.00 | 1.29 | 0.11 | 1.39 | 0.09 | 1.49 | 0.04 |
| **Isovalerate** | 1.00 | | 1.00 | | 1.00 | | 1.57 | 0.04 | 7.39 | 0.00 | 16.9 | 0.00 |
| **Caproate** | 1.20 | 0.39 | 1.42 | 0.24 | 1.38 | 0.38 | 1.08 | 0.96 | 1.51 | 0.10 | 2.34 | 0.02 |
| **Caprylate** | 1.35 | 0.07 | 1.56 | 0.06 | 2.05 | 0.00 | 2.11 | 0.00 | 2.20 | 0.00 | 2.78 | 0.00 |
| **5-dodecenoate** | 1.00 | | 1.21 | 0.39 | 1.63 | 0.00 | 1.30 | 0.08 | 1.57 | 0.00 | 1.99 | 0.00 |
| **Myristoleate** | 3.15 | 0.01 | 4.61 | 0.01 | 7.36 | 0.00 | 3.10 | 0.00 | 4.90 | 0.00 | 6.06 | 0.00 |
| **Arachidonate** | 2.13 | 0.01 | 2.31 | 0.00 | 2.91 | 0.00 | 1.37 | 0.01 | 1.59 | 0.03 | 1.76 | 0.00 |
| **Ethanolamine** | 1.68 | 0.05 | 1.72 | 0.01 | 2.30 | 0.00 | 0.91 | 0.53 | 1.05 | 0.83 | 1.25 | 0.14 |
| **Glycerophosphorylcholine** | 1.44 | 0.10 | 1.80 | 0.03 | 2.00 | 0.02 | 0.92 | 0.03 | 0.94 | 0.07 | 0.98 | 0.45 |
| **Myo-inositol** | 1.02 | 0.73 | 1.03 | 0.21 | 1.08 | 0.04 | 1.18 | 0.07 | 1.08 | 0.43 | 1.07 | 0.92 |

| | | | | | | | | | | | | |
|---|---|---|---|---|---|---|---|---|---|---|---|---|
| **1-palmitoyl-glycerophosphocholine** | 0.93 | 0.99 | 1.46 | 0.16 | 1.08 | 0.86 | 1.69 | 0.02 | 1.59 | 0.03 | 1.04 | 0.12 |
| **1-stearoyl-glycerophosphocholine** | 0.93 | 0.88 | 1.45 | 0.17 | 1.19 | 0.71 | 1.75 | 0.01 | 1.75 | 0.01 | 1.42 | 0.11 |
| **1-oleoyl-glycerophosphocholine** | 0.84 | 0.81 | 1.41 | 0.20 | 1.12 | 0.82 | 1.78 | 0.02 | 1.60 | 0.04 | 1.43 | 0.18 |
| **1-arachidon-oylglycerophosphocholine** | 0.71 | 0.65 | 1.81 | 0.12 | 1.18 | 0.64 | 1.61 | 0.04 | 1.33 | 0.17 | 1.15 | 0.51 |
| **Xanthine** | 1.17 | 0.00 | 1.23 | 0.00 | 1.34 | 0.00 | 0.93 | 0.50 | 0.89 | 0.30 | 0.90 | 0.32 |
| **Xanthosine** | 1.02 | 0.58 | 1.16 | 0.01 | 1.25 | 0.01 | 0.92 | 0.10 | 0.95 | 0.39 | 0.83 | 0.02 |
| **Hypoxanthine** | 1.69 | 0.00 | 2.06 | 0.00 | 2.42 | 0.00 | 1.00 | | 1.00 | | 1.00 | |
| **Inosine** | 1.16 | 0.01 | 1.10 | 0.04 | 1.08 | 0.10 | 0.78 | 0.09 | 0.77 | 0.16 | 0.58 | 0.00 |
| **2'-deoxyinosine** | 0.94 | 0.12 | 0.89 | 0.02 | 0.81 | 0.00 | 1.00 | | 1.00 | | 1.00 | |
| **Adenine** | 0.93 | 0.30 | 0.94 | 0.13 | 0.86 | 0.09 | 1.02 | 0.80 | 0.85 | 0.14 | 0.84 | 0.09 |
| **Adenosine** | 0.03 | 0.00 | 0.05 | 0.00 | 0.04 | 0.00 | 1.00 | | 1.00 | | 1.00 | |
| **Adenosine 3'-monophosphate** | 1.00 | | 1.00 | | 1.00 | | 1.44 | 0.02 | 1.51 | 0.01 | 1.36 | 0.03 |
| **Adenosine 5'-monophosphate** | 2.19 | 0.00 | 2.48 | 0.00 | 1.74 | 0.01 | 1.17 | 0.03 | 1.34 | 0.00 | 1.39 | 0.00 |
| **Guanine** | 1.98 | 0.00 | 2.15 | 0.00 | 2.21 | 0.00 | 0.65 | 0.07 | 0.51 | 0.03 | 0.59 | 0.04 |
| **Guanosine** | 0.91 | 0.26 | 0.96 | 0.47 | 0.89 | 0.23 | 1.44 | 0.01 | 1.19 | 0.21 | 1.10 | 0.44 |
| **2'-Deoxy-guanosine** | 0.73 | 0.03 | 0.70 | 0.01 | 0.55 | 0.01 | 1.00 | | 1.00 | | 1.00 | |
| **1-methyl-guanosine** | 0.93 | 0.49 | 0.97 | 0.83 | 0.94 | 0.61 | 1.00 | | 1.00 | | 1.00 | |
| **Urate** | 0.76 | 0.16 | 0.71 | 0.03 | 0.63 | 0.02 | 0.98 | 0.90 | 0.94 | 0.50 | 0.84 | 0.17 |
| **Uridine** | 0.99 | 0.86 | 1.03 | 0.47 | 0.98 | 0.75 | 1.20 | 0.18 | 1.25 | 0.14 | 1.30 | 0.02 |
| **Nicotinamide** | 1.06 | 0.23 | 1.25 | 0.02 | 1.04 | 0.76 | 1.13 | 0.32 | 1.56 | 0.02 | 1.34 | 0.07 |
| **Nicotinamide adenine dinucleotide** | 0.25 | 0.00 | 0.25 | 0.00 | 0.25 | 0.00 | 0.26 | 0.00 | 0.23 | 0.00 | 0.23 | 0.00 |
| **Adenosine 5'diphosphoribose** | 0.77 | 0.06 | 0.77 | 0.06 | 0.77 | 0.06 | 1.03 | 0.86 | 0.74 | 0.05 | 0.63 | 0.01 |
| **Trigonelline** | 0.37 | 0.00 | 0.27 | 0.00 | 0.27 | 0.00 | 1.48 | 0.33 | 1.04 | 0.99 | 1.24 | 0.43 |

| | | | | | | | | | | | | |
|---|---|---|---|---|---|---|---|---|---|---|---|---|
| **Pantothenate** | 0.97 | 0.77 | 1.04 | 0.54 | 0.91 | 0.40 | 1.18 | 0.02 | 0.99 | 0.81 | 1.05 | 0.36 |
| **Hippurate** | 1.04 | 0.29 | 1.05 | 0.21 | 1.07 | 0.04 | 1.04 | 0.48 | 1.05 | 0.36 | 1.00 | 0.93 |
| **Glycolate** | 0.60 | 0.09 | 1.17 | 0.41 | 0.72 | 0.26 | 1.56 | 0.04 | 1.26 | 0.28 | 1.22 | 0.25 |
| **Phenol sulfate** | 1.02 | 0.67 | 0.99 | 0.86 | 1.10 | 0.01 | 0.95 | 0.68 | 1.06 | 0.60 | 1.06 | 0.48 |
| **Daidzein** | 1.00 | | 1.00 | | 1.00 | | 1.18 | 0.02 | 1.31 | 0.00 | 1.28 | 0.00 |
| **Genistein** | 1.00 | | 1.00 | | 1.00 | | 1.12 | 0.00 | 1.04 | 0.11 | 1.08 | 0.07 |
| **Erythritol** | 0.97 | 0.68 | 0.94 | 0.46 | 1.01 | 0.87 | 1.11 | 0.00 | 1.13 | 0.07 | 1.04 | 0.64 |

34

**Table S2:** An overview of the average feed intake per gerbil per day and the average weight gain per gerbil per week in the non-infected control groups (Control) and in gerbils experimentally infected with *Helicobacter suis* (Infected), fed a non-supplemented diet (Std), a diet supplemented with 5% glutamine (Gln) or a diet supplemented with 0.8% glutathione (GSH). Weight is expressed in grams ± standard deviation.

| | **Diet** | **Estimated average feed intake per gerbil per day** | **Average weight gain per gerbil per week** |
|---|---|---|---|
| **Control** | **Std** | 3.4 | 1.50 ± 0.16 |
| | **Gln** | 3.9 | 1.65 ± 0.12 |
| | **GSH** | 3.8 | 1.08 ± 0.21 |
| **Infected** | **Std** | 3.6 | 1.43 ± 0.31 |
| | **Gln** | 3.7 | 1.53 ± 0.43 |
| | **GSH** | 3.9 | 1.33 ± 0.30 |

**TABLE S3:** Overall inflammation in the stomach of gerbils experimentally infected with *Helicobacter suis*. [1]The gerbils received either a standard diet, a standard diet supplemented with 5% glutamine or a standard diet supplemented with 0.8% glutathione. [2]Inflammation scores: 0 = no inflammation; 1 = mild diffuse infiltration of lymphocytes or presence of one small aggregate of inflammatory cells; 2 = moderate diffuse infiltration and/or the presence of 2-4 inflammatory aggregates; 3 = marked diffuse infiltration and/or the presence of at least 5 inflammatory aggregates. [3]Displayed are the number of animals out of ten animals in each infected group with a specific overall inflammation score.

| **Groups[1]** | **Inflammation score antrum** | | | | **Inflammation score corpus** | | | |
|---|---|---|---|---|---|---|---|---|
| | **0[2]** | **1** | **2** | **3** | **0** | **1** | **2** | **3** |
| **Standard** | 0[3] | 0 | 4 | 6 | 7 | 3 | 0 | 0 |
| **Glutamine** | 1 | 2 | 4 | 3 | 8 | 2 | 0 | 0 |
| **Glutathione** | 7 | 3 | 0 | 0 | 9 | 1 | 0 | 0 |
